# Typical US, CT and MR Findings in Multiple Biliary Hamartomas

**DOI:** 10.5334/jbr-btr.1249

**Published:** 2017-01-23

**Authors:** Julien Van Dorpe, Maxime Hackx

**Affiliations:** 1 CHU Tivoli, BE; 2 CHR Haute Senne, BE

**Keywords:** US, CT, MR, liver, hamartoma

A 38-year-old woman without known malignancy was referred to the Department of Radiology for acute epigastric pain. An abdominal US was first performed that revealed multiple small hyperechoic liver lesions with comet-tail echoes (Figure 1). An abdominal CT was performed that revealed multiple small hypodense liver lesions with no enhancement after intravenous administration of iodine contrast (Figure 2). On the basis of these US and CT findings, the diagnosis of multiple biliary hamartomas (MBHs) was suggested. A month later, an abdominal MRI was performed revealing multiple small liver lesions hyperintense on T2-weighted images and hypointense on T1-weighted image with no enhancement after intravenous administration of gadolinium. In addition, MR cholangiography showed multiple small cystic liver lesions scattered through both liver lobes, with normal appearances of the intrahepatic and extrahepatic bile ducts. There was no communication between the lesions and the draining bile ducts (Figure 3). As the patient had no known malignancy, and as the US, CT and MR findings were typical of MBHs, no histologic confirmation was requested.

**Figure 1:**
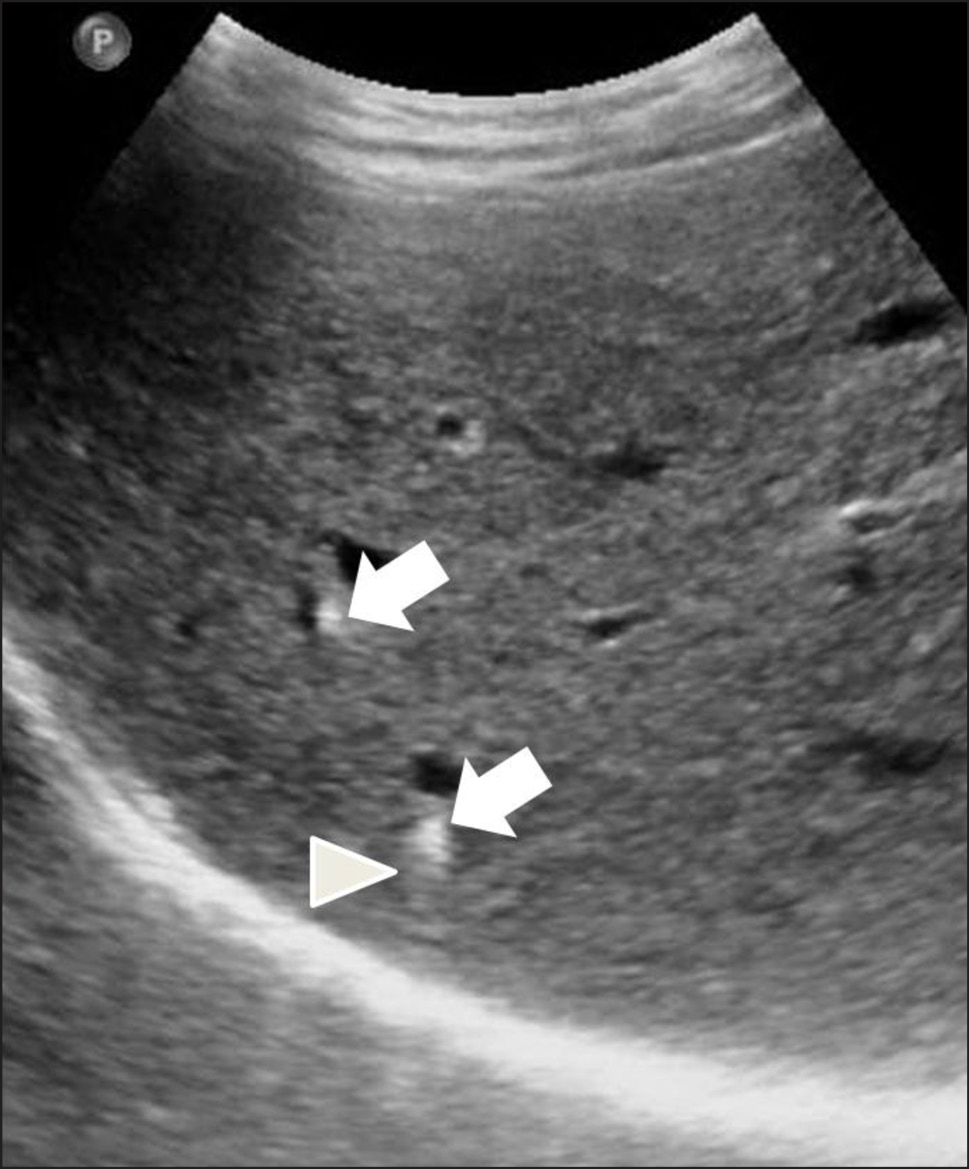
US image of the liver showing hyperechoic liver lesions (arrows) with comet-tail echoes (arrowhead).

**Figure 2:**
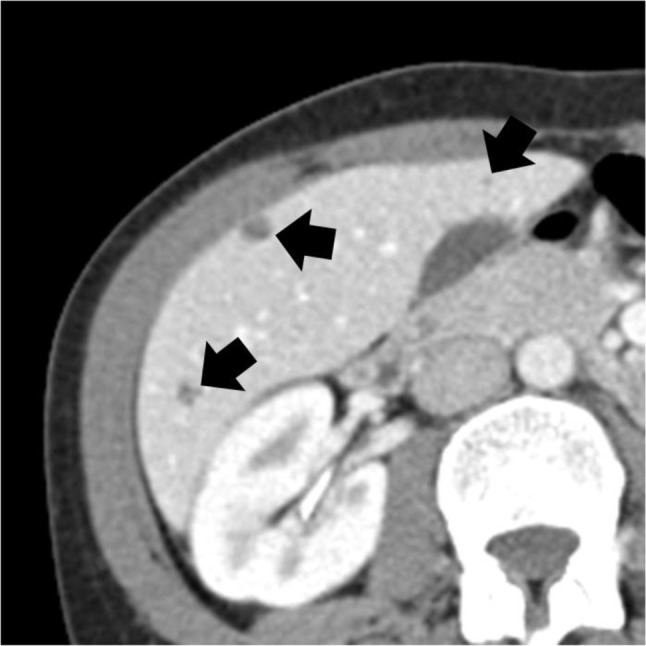
Enhanced axial CT image of the abdomen showing multiple small non-enhancing hypodense liver lesions (arrows).

**Figure 3:**
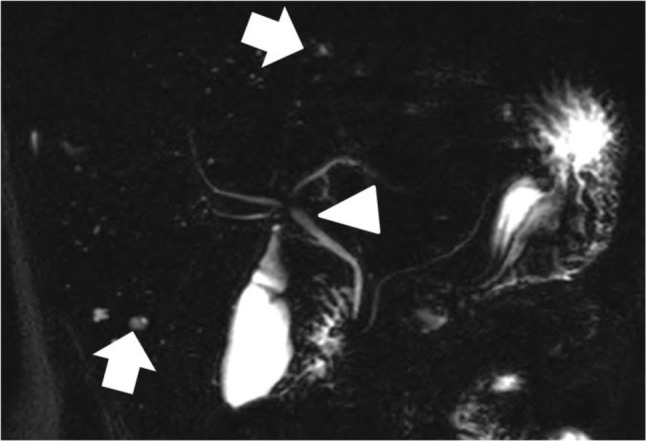
MR cholangiography showing multiple small cystic liver lesions scattered through both liver lobes (arrows), with normal appearances of the intrahepatic and extrahepatic bile ducts (arrowhead).

Imaging manifestations of MBHs are known to be various and therefore confusing, with biopsy still remaining the standard for definitive diagnosis [1]. Some authors, however, suggested that biopsy should only be performed in patients with a history of primary malignancy or with atypical imaging findings, and that the diagnosis of MBHs might thus be considered by radiologists when typical imaging findings are found (i.e. multiple small-comet tails echoes on US, multiple small hypodense lesions scattered throughout the liver with no enhancement on CT, and cystic appearance with normal extra- and intrahepatic bile ducts on MR and MRCP) [1].

## References

[1] Zheng RQ, Zhang B, Kudo M, Onda H, Inoue T (2005). Imaging findings of biliary hamartomas. World J Gastroenterol.

